# Setting a benchmark for resource utilization and quality of care in patients undergoing transcatheter aortic valve implantation in Europe—Rationale and design of the international BENCHMARK registry

**DOI:** 10.1002/clc.23711

**Published:** 2021-09-09

**Authors:** Gemma McCalmont, Eric Durand, Sandra Lauck, Douglas F. Muir, Mark S. Spence, Mariuca Vasa‐Nicotera, David Wood, Francesco Saia, Nicolas Chatel, Claudia M. Lüske, Jana Kurucova, Peter Bramlage, Derk Frank

**Affiliations:** ^1^ Cardiology Department James Cook University Hospital Middlesbrough UK; ^2^ Department of Cardiology, FHU CARNAVAL Normandie University, UNIROUEN Rouen France; ^3^ Center for Heart Valve Innovation, St Paul's Hospital University of Vancouver Vancouver British Columbia Canada; ^4^ Cardiology Department, Royal Victoria Hospital Belfast UK; ^5^ Cardiology Department Frankfurt University Frankfurt Germany; ^6^ Department of Cardiology University of Bologna Bologna Italy; ^7^ Edwards Lifesciences Nyon Switzerland; ^8^ Institute for Pharmacology and Preventive Medicine Cloppenburg Germany; ^9^ Edwards Lifesciences Prague Czech Republic; ^10^ Department of Internal Medicine III (Cardiology, Angiology and Intensive Care Medicine) University Clinical Center Schleswig‐Holstein (UKSH) Kiel Germany; ^11^ German Center for Cardiovascular Research, partner site Hamburg/Kiel/Lübeck Kiel Germany

**Keywords:** aortic stenosis, quality of care, clinical practice, registry, transcatheter aortic valve implantation, TAVI

## Abstract

**Background:**

The use of transcatheter aortic valve implantation (TAVI) for treating aortic stenosis (AS) has increased exponentially in recent years. Despite the availability of clinical practice guidelines for the management of valvular heart disease, disparities in quality of care (QoC) for TAVI patients remain widespread across Europe. Tailored QoC measures will help to reduce resource utilization and improve patient outcomes without compromising patient safety. Using a clear set of QoC measures, the BENCHMARK registry aims to document the progress that can be achieved if such tailored QoC measures are implemented.

**Methods:**

The BENCHMARK registry (BENCHMARK) is a non‐interventional, multicenter registry in patients with severe symptomatic AS undergoing TAVI with a 1‐ and 12‐months follow‐up. BENCHMARK will be conducted at 30 centers across Europe and will enroll a total of 2400 consecutive TAVI patients. Patients suffering from severe symptomatic AS who undergo TAVI with a balloon‐expandable transcatheter aortic valve will be included. The registry will comprise four phases: (1) a retrospective baseline evaluation phase; (2) an education phase; (3) an implementation phase; and (4) a prospective effect documentation phase (prospective phase). The registry's primary objectives are to reduce the length of hospital stay and accelerate the post‐procedural patient recovery pathway, but without compromising safety. The study started in April 2021 and has an estimated completion date of May 2023.

**Discussion:**

BENCHMARK will establish QoC measures to reduce resource utilization, intensive care unit bed occupancy, and overall length of hospitalization with uncompromised patient safety post‐TAVI (ClinicalTrials.gov Identifier: NCT04579445).

## Abstract 2

**Highlights**

- The BENCHMARK registry is a non‐interventional, multicenter international registry.
- A total of 2400 patients with severe AS will be enrolled at 30 European centers.
- The registry is designed to be partly retrospective and partly prospective.
- The BENCHMARK registry will help to improve TAVI QoC standards across Europe.

## 1 INTRODUCTION

Transcatheter aortic valve implantation (TAVI) has emerged as standard of care for patients suffering from severe, symptomatic aortic stenosis (AS), irrespective of the level of surgical risk.^1^, ^2^, ^3^, ^4^, ^5^ Since its introduction almost two decades ago,^6^ an evolving understanding of patient and prosthesis selection, increased peri‐procedural expertise, and advances in valve technology have contributed to improved outcomes and patient access to TAVI.^7^

As a consequence of TAVI indications expanding to younger and lower‐risk patients, there has been a clear shift in interest toward reducing healthcare resource utilization, and identifying factors likely to predict potential futility of the procedure^5^, ^8^, ^9^, ^10^ For example, a minimalist approach to help reduce procedure waiting times, resource use and costs, length of hospital stay, and staff workload.^11^, ^12^ Local programs that incorporate procedural algorithms to simplify the TAVI care pathway have been developed by many centers.^8^, ^12^, ^13^, ^14^, ^15^ However, the quality of care (QoC) for TAVI patients across Europe remains highly variable, particularly in terms of effective screening and patient discharge/follow‐up management.^16^, ^17^ A unified strategy is warranted to ensure that all patients receive consistent pre‐, peri‐ and post‐TAVI care and optimized outcomes.^18^

To achieve standardized QoC, some centers of excellence have introduced a dedicated coordinator or TAVI nurse to ensure a streamlined care pathway for all patients.^19^, ^20^ The TAVI coordinator/nurse helps to manage the procedural program for individual patients while maintaining seamless communication with the Heart Team throughout the patient care journey, making the process more streamlined.^19^ One of the TAVI coordinator's roles is to ensure that all relevant screening results (e.g., echocardiography, computed tomography [CT] imaging scans, coronary angiogram, blood tests, etc.) are distributed to all members of the Heart Team promptly to inform patient discussion and to prevent delays in Heart Team recommendations.^5^ Lauck et al.^19^ endorse this coordinated, streamlined approach to reduce the length of hospital stays and appropriately distribute healthcare resources. The recent multicenter European Feasibility and Safety of Early Discharge After Transfemoral TAVI (FAST‐TAVI)^21^, ^22^ and Vancouver 3M (multidisciplinary, multimodality, but minimalist) transfemoral transcatheter aortic valve replacement (TAVR) studies^17^, ^23^, ^24^, ^25^ have shown that adhering to a minimalist strategy, with optimized criteria for risk assessment and patient discharge management, results in a more efficient care pathway, thus reducing the length of hospital stay and enabling a safe and timely discharge for patients. Furthermore, these studies have gained added importance recently due to the unprecedented stress on healthcare resources caused by the Coronavirus disease (Covid‐19) pandemic.^19^, ^26^

A consistent pre‐, peri‐, and post‐procedural management strategy is essential to improve QoC for TAVI patients. Implementing a tailored set of QoC measures as a benchmark for best practice will further reduce resource utilization, intensive care unit (ICU) bed occupancy, and overall length of hospitalization without compromising patient safety post‐TAVI. The BENCHMARK registry aims to document the progress that can be achieved in clinical practice if consistent QoC measures are initiated in TAVI centers and the rationale for this study is supported by the FAST TAVI, the French FAST TAVI 1 and Vancouver 3M TAVR studies.^13^, ^14^, ^17^, ^21^, ^22^

## 2 METHODS/DESIGN

The BENCHMARK registry (ClinicalTrials.gov Identifier: NCT04579445) is a non‐interventional, multicenter, international registry that will enroll patients with severe symptomatic AS undergoing transfemoral (TF) TAVI at 30 centers across Europe (Austria, Czech Republic, France, Germany, Italy, Romania, Spain). Each participating center should have a TAVI coordinator in place. The registry will be conducted according to the European Medical Device Regulations and International Organization for Standardization (ISO 14155:2020) and the ethical principles originating from the Declaration of Helsinki. All participants will provide written informed consent before enrolling in the registry. The protocol and patient informed consent forms will be approved by the local Institutional Review Board (IRB)/Independent Ethics Committee (IEC) at each center prior to initiation of the registry.

### 2.1 Study design

**FIGURE 1:**
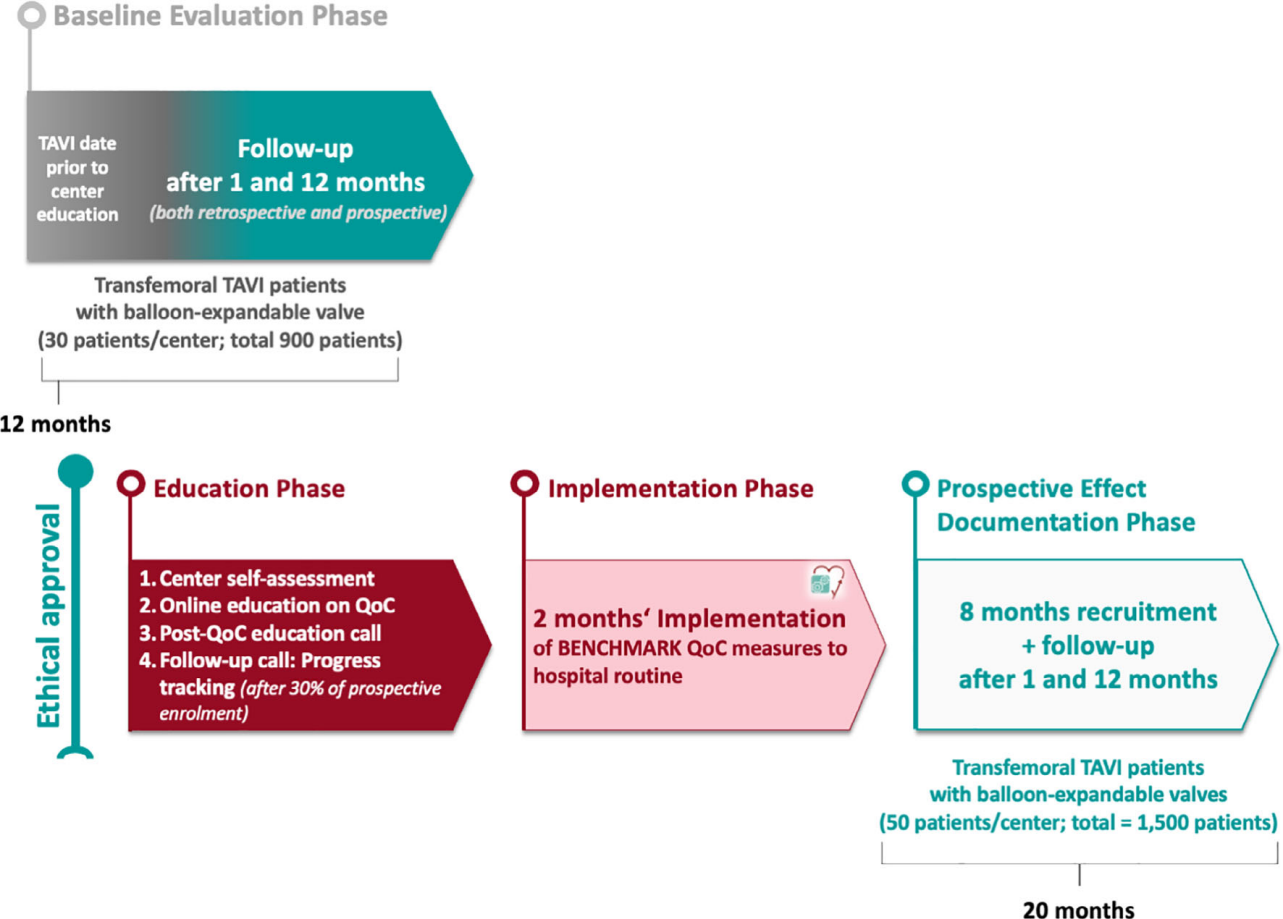
Registry design (retrospective baseline evaluation phase, education phase, implementation phase and prospective effect documentation phase). The registry comprises four phases: (1) A retrospective baseline evaluation phase—this phase is the current status quo and includes documentation of treatment pathways and endpoints of “routine” patients. (2) An education phase, which phase provides education on the BENCHMARK quality of care measures and involves the self‐assessment of centers. (3) An implementation phase, which aims to improve routine hospital quality of care measures. (4) A prospective effect documentation phase—this phase documents the impact of implemented BENCHMARK quality of care measures on treatment pathways, outcomes, safety, and resource utilization. Note, each participating center should have a non‐physician TAVI coordinator in place. QoC, quality of care; TAVI, transcatheter aortic valve implantation

**FIGURE 2:**
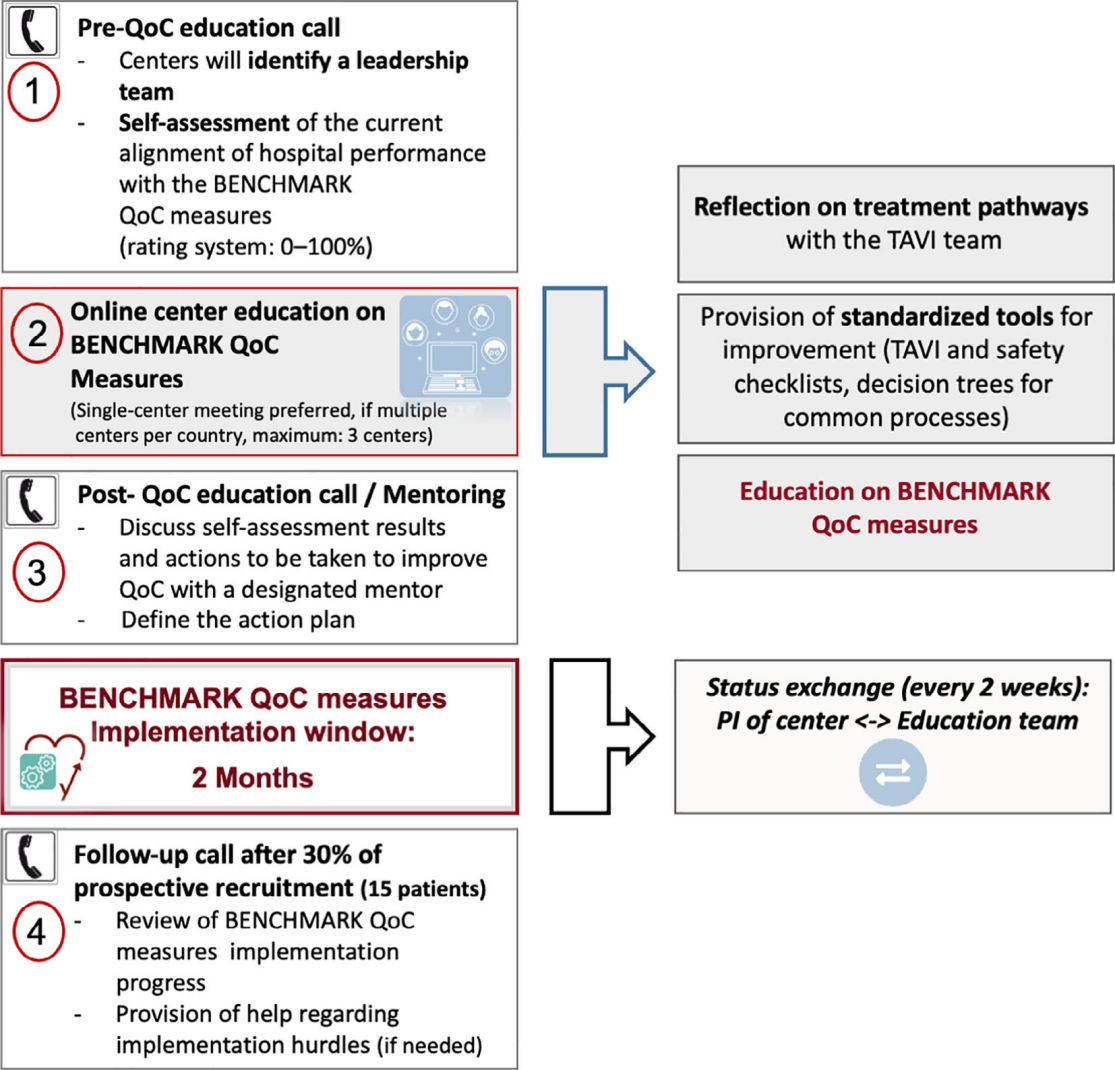
Education pathway (BENCHMARK QoC measures). Pathway stages 1–4: (1) center self‐assessment is performed prior to the online education seminar; (2) online center education on BENCHMARK QoC measures will be arranged by the education team (including the local steering committee member and/or the PI team and the registry management team). A minimum of 3 TAVI Team members, having been identified as a leadership team (TAVI coordinator and further staff members per site), will attend the seminar; (3) a post‐QoC education call to discuss the self‐assessment results and write a final action plan with the leadership team will be arranged 1 week after the education seminar; and (4) a follow‐up call will be arranged to review center progress on BENCHMARK QoC implementation. PI, principal investigator; QoC, quality of care

The BENCHMARK registry will document the effect of introducing tailored BENCHMARK QoC measures (Table 1) into TAVI centers using four distinct phases: Retrospective baseline evaluation phase, education phase, implementation phase, and prospective effect documentation phase (Figure 1). For the baseline evaluation phase (retrospective phase), each center will retrospectively document 30 consecutive patients (*N* = 900) undergoing transfemoral TAVI with a balloon expandable transcatheter aortic valve before introducing the BENCHMARK QoC measures. The TAVI procedure and patient discharge within this phase must have been performed prior to the first educational phone call. For each patient, follow‐up data after 1 and 12 months will be recorded. In the education phase, each center will identify a defined leadership team (i.e., multidisciplinary heart team) to undergo online education on the BENCHMARK QoC measures and best practices. A detailed description of the education phase is shown in Figure 2. During the Implementation Phase, consisting of a 2‐month time window, each center will introduce the tailored QoC measures into their hospital routine. Follow‐up calls will be arranged between the BENCHMARK education team and each center every 2 weeks to offer assistance with regard to implementation progress. Finally, each center will prospectively enroll 50 consecutive patients (*N* = 1500) undergoing TAVI after completing the education and implementation phases. This prospective phase is estimated to be 20 months duration overall, with up to 8 months for patient recruitment and 12 months follow‐up for the last patient included.

**TABLE 1:** BENCHMARK QoC measures

BENCHMARK QoC measures	Implementation description
1. Education of patient and family	The patient, and if the patient is not independent, at least one family member or carer is involved in the patient‐education, and discharge provision is discussed with them prior to the procedure.
2. Education and alignment of the internal team (medical and paramedical)	At least one joint meeting per year is organized to educate all staff involved in the diagnostics, post‐procedural care and intervention of patients with severe AS.
3. Determination of an anticipated discharge date at admission based on pre‐procedural risk stratification and scheduling of post‐procedural diagnostics accordingly	Anticipated discharge date is determined at admission, and post‐procedural diagnostics are scheduled accordingly.
4. Echo‐ or angiographic check at the end of procedure is performed to confirm proper closing of access site and proper management of all complications is done immediately	Echo‐ or angiographic check is performed in the hybrid room, and even minor vascular complications are treated immediately.
5. Early mobilization of the patient	Mobilization of the patient with the help of a nurse is done 4–6 h after the intervention in absence of complications.
6. Using a decision tree to determine the need for new pacemaker implantation without increasing hospital stay	Detailed decision tree with required diagnostic work‐up is in place and has been followed for each patient.
7. Daily visit to patient by implanter and interaction with rest of the team	At least one daily visit of the patient is being done by a TAVI implanter (team) during hospitalization from the day before the intervention up to patient discharge.
8. Criteria based discharge	Early discharge decision protocol (or checklist) is in place and is followed for each patient.

*Note*: The 8 BENCHMARK QoC measures are rated 0%–100%.

Abbreviations: AS, aortic stenosis; Echo, echocardiogram; PPM, permanent pacemaker implantation; QoC, quality of care.

### 2.2 Patients

A total number of 2400 patients aged ≥18 years old with symptomatic AS who undergo TF TAVI with a balloon‐expandable transcatheter aortic valve will be included. Approximately 900 consecutive patients will be documented in the retrospective baseline evaluation phase (30 per center) and 1500 patients in the prospective phase (50 per center). The sample size estimate is based on the ability to discriminate any changes in outcomes between the retrospective baseline evaluation phase and the prospective effect documentation phase.

All patients irrespective of transcatheter valve type or access route, will be documented in an electronic case report form (eCRF) based patient logbook. A defined core data set will be collected for all patients (mortality, stroke, time of discharge/length of hospital stay, readmission).

### 2.3 Inclusion and exclusion criteria

In the baseline evaluation phase, inclusion criteria included patients of at least 18 years of age, consecutive patients with severe symptomatic AS who underwent transfemoral TAVI with a balloon‐expandable transcatheter aortic valve prior to the center education on BENCHMARK QoC measures, and if the patient was scheduled to undergo 30‐day and 12‐month follow‐up visits. Patients were excluded from the Baseline Evaluation Phase if their data was incomplete respect to the aims of the registry (length of hospital stay, time on the ICU), they did not provide informed consent, or were pregnant (Table 2).

**TABLE 2:** BENCHMARK inclusion/exclusion criteria

Inclusion criteria	Exclusion criteria
*Baseline evaluation phase*	
Patient is at least 18 years old Consecutive patients with severe symptomatic AS who underwent transfemoral TAVI with a balloon‐expandable transcatheter aortic valve *prior* to the center education on BENCHMARK Quality of Care measures (prior to the pre‐Quality of Care education call) Patient is or was scheduled to undergo 30 days and 12 months follow‐up visits (30 days and 12 month follow‐up: hospital visit or phone call)	Patients with largely incomplete data with respect to the aims of the project. Patients without signed informed consent/data protection statement (unless otherwise agreed by the local Institutional Review Board/Independent Ethics Committee) Pregnancy at time of the TAVI
*Prospective effect documentation phase*	
Patient is at least 18 years old Consecutive patients with a diagnosis of severe symptomatic AS admitted for transfemoral TAVI with a balloon‐expandable transcatheter aortic valve *after* center education on the BENCHMARK Quality of Care measures/*after* the center has passed the Implementation Phase. Patient is scheduled to attend follow‐up visits at the center 30 days and 12 months after the procedure (both visits taking place in the hospital)	Patients without signed informed consent/data protection statement (according to requirements of local Institutional Review Board/Independent Ethics Committee) Pregnancy at time of the TAVI

Abbreviations: AS, aortic stenosis; TAVI, transcatheter aortic valve implantation.

In the Prospective Effect Documentation Phase, the inclusion criteria were patients of at least 18 years of age, consecutive patients with a diagnosis of severe symptomatic AS admitted for transfemoral TAVI with a balloon‐expandable transcatheter aortic valve after center education on BENCHMARK QoC measures/after the center had passed the Implementation Phase, and the patient is scheduled to attend follow‐up visits at the center 30 days and 12 months after the procedure. Patients were excluded from the Prospective Effect Documentation Phase if they did not provide informed consent or were pregnant (Table 2 **)**.

Patients undergoing valve‐in‐valve procedures or repeat TAVI will not be included in the registry.

### 2.4 Data collection

Clinical outcome data collected will be based on the center's standard of care for TAVI. Data will be collected according to the timetable set out in Table 3, and include physical assessments, medical history and symptoms, diagnostics, electrocardiography (ECG), echocardiography (Echo), hospitalization and procedural duration, safety parameters, QoL measures, satisfaction surveys, as well as resource utilization parameters. Data will be captured in an eCRF by either a study nurse or physician, and the registry sponsor will check all data for plausibility and completeness.

**TABLE 3:** Data collection schedule

	Baseline evaluation phase (retrospective documentation phase)					Effect documentation of BENCHMARK QoC measures (prospective enrollment phase)				
	Screening/baseline	TAVI	Discharge	30 days visit *Visit or call* (±1 week)	12 months visit *Visit or call* (±3 weeks)	Screening/baseline	TAVI	Discharge	30 days visit (±1 week)	12 months visit (±3 weeks)
Signed informed consent	X^a^					X^b^				
Inclusion/exclusion criteria	X					X				
Referral management	X					X				
Treatment decision	X					X				
Tasks, being impacted by the coordinator								X		
Demographics	X					X				
ECG and Echo	X	X	X	X^c^	X^c^	X	X	X	X	X
Comorbidities	X					X				
Symptoms and social characteristics	X					X				
Risk score (Euro Score II)	X					X				
Frailty, mental, social status	X					X				
Quality of life (TASQ)						X		X	X	X
Procedural details (device success)		X					X			
Length of hospital stay			X					X		
Safety and efficacy variables (VARC‐2)^27^			X	X	X			X	X	X
Rehospitalization data				X	X				X	X
Adverse event reporting		X	X	X	X		X	X	X	X
BENCHMARK QoC measures (center and patient based)								X		
Patient/physician/coordinator/nursing staff satisfaction			X^d^					X		
Self‐reported working hours per patient (physician, coordinator, nursing staff)								X		

Abbreviations: ECG, electrocardiogram; Echo, echocardiogram; IRB, institutional review board; TAVI, transcatheter aortic valve implantation; QoC, quality of care; TASQ, Toronto Aortic Stenosis Quality of Life Questionnaire; VARC‐2, Valve Academic Research Consortium‐2 consensus document.

^a^ The TAVI and discharge visits of patients being eligible for retrospective documentation must have been documented prior to site's education on BENCHMARK QoC measures. Need for consent form according to local IRB requirements.

^b^ Consent needs to be given prior to the TAVI procedure.

^c^ 30 day and 12 months follow‐up (retrospective phase) either hospital visit (preferred) or performed via phone call (no ECG/Echo will be available for this option).

^d^ Optional: Survey could be administered to retrospective patients in combination with consent form (e.g., via postal way).

### 2.5 Registry objectives

The primary objective of the BENCHMARK registry is to document the effect of introducing tailored BENCHMARK QoC measures into TAVI centers to (1) decrease length of hospital stay; and (2) reduce the need for ICU capacity. Secondary objectives will be to streamline diagnostics, minimize staff workload allowing timely delegation of responsibilities, ascertain uncompromised patient safety post‐TAVI (VARC‐2 defined early and time‐related valve safety), improve patient quality of life (QoL), patient and staff satisfaction, and improve the implementation of BENCHMARK QoC measures by each center over time. A full description of the primary and secondary objectives of the BENCHMARK registry is provided in Table 4.

**TABLE 4:** BENCHMARK registry objectives

Primary objectives	
Reduction in length of hospital stay	To reduce the length of hospital stay (door to needle, needle to door, overall stay) in order to reduce overall costs based on hospitalization, and to enable early return to normal life: Separate single hospitalization for diagnostics Door to needle (days) Needle to door (days) Timely discharge to home, rehabilitation center or other institution Economic effect assessment associated with hospitalization changes after implementation of BENCHMARK QoC measures
Reduction of the need for ICU capacity	To accelerate the post‐procedural patient care recovery pathway: Minimize/eliminate time spent in the recovery room, ICU, CCU or IMC and prioritize a rapid return to general ward, in order to reduce the level of invasiveness of post procedural care (e.g., with regard to COVID‐19): Time spent in the recovery room (hours) Time spent on the ICU (hours) Time spent on the CCU (CCU) (hours) Time spent on the IMC (hours/days) Time spent on the general ward (hours/days)

Secondary objectives	
Streamlining of diagnostic and procedural times	To streamline diagnostics to avoid duplication of diagnostic measures and to tighten schedules: Proportion of repeated diagnostic procedures Patient status assessment: physician versus nurse/coordinator To reduce the procedural time without compromising patient safety post TAVI: Time between incision and sheath removal (min) Time between entering and leaving the OR/catheterization laboratory/hybrid room (min)
Reduce the use of human resources, patient flow optimization	To reduce staff workload and to perform timely delegation of responsibilities to minimize the physiciansˈ workload: Working hours per patient collected from physicians, nursing staff, medical technical assistant, TAVI coordinator Economic assessment of working hours needed To increase number of patients with severe AS undergoing TAVI per day to reach higher hospital efficiency: Mean number of interventions performed per day/per week (on center level)
Maintenance of patient safety	To ascertain uncompromised safety post TAVI, by successful TAVI performance and reduced procedural complications: Device success and procedural complications: Device success Conduction disturbance Pacemaker implantations Conversion to open heart surgery Moderate or severe paravalvular leak Safety at 30 days and 12 months (VARC‐2)^27^: Mortality (cardiac/non‐cardiac) Stroke/TIA Major vascular complications PPM (at 30 days) (Cardiac) hospital readmission Number and type of adverse events: Economic assessment with regard to avoided TAVI associated complications
Improvement of patient QoL as well as patient and staff satisfaction	To improve patient QoL and satisfaction, to achieve relief of staff workload, and to make sure that improved structures are translated into patient relevant outcomes: Patient QoL assessment (TASQ) Patient satisfaction (tailored survey) Physician/Coordinator satisfaction (tailored survey)
Improved QoC by implementation of BENCHMARK QoC measures	To increase the implementation progress of the BENCHMARK QoC measures per center over time to document the structural achievement in the patient pathway (center self‐assessment on a monthly basis, patient based assessment in the eCRF): Rating of 8 BENCHMARK QoC measures (rating 0%–100%)

Abbreviations: AS, aortic stenosis; CCU, Coronary Care Unit; eCRF, electronic case report form; TAVI, transcatheter aortic valve implantation; ICU, intensive care unit; IMC, immediate care; OR, operating room; PPM, permanent pacemaker implantation; Pts, patients; QoC, quality of care; QoL, quality of life; TASQ, Toronto Aortic Stenosis Quality of Life Questionnaire; VARC‐2, Valve Academic Research Consortium‐2 consensus document.

### 2.6 Statistical analysis

Statistical analysis will be performed for the total registry population as well as for defined subgroups if applicable. Continuous variables will be presented as mean ± SD or median with interquartile range, and categorical variables (e.g., gender) will be reported as frequencies and percentages. The Kolmogorov–Smirnov test may be used to test for normal distribution. For comparison, χ^2^ test or Fisher's exact test may be used for categorical variables, and *t* test or Mann‐Whitney U test for continuous variables. Linearized rates and actuarial probability statistics may be used where appropriate for adverse event reporting. Kaplan–Meier analysis may be performed for survival and safety outcomes. All statistical analyses will be performed using IBM SPSS Statistics version 24 (IBM, Armonk, New York) or R Core Team (https://www.R-project.org/).

## 3 DISCUSSION

The BENCHMARK registry has been designed to document the effect of introducing tailored BENCHMARK QoC measures into TAVI centers. Analysis of the data gathered may provide additional insights to further refine and improve QoC measures and best practices for the effective management of patients with severe AS. The knowledge acquired from the BENCHMARK registry dataset will help to standardize care pathways and treatment outcomes for TAVI patients across Europe.

### 3.1 Studies that support the BENCHMARK registry

Results from the European FAST‐TAVI study support the BENCHMARK registry and show that the use of a pre‐defined set of QoC measures can lead to reduced use of medical resources, improved QoL, and optimized patient outcomes.^21^, ^22^ FAST‐TAVI is a real‐world, observational, prospective trial designed to assess early discharge feasibility and safety after transfemoral TAVI (TF‐TAVI).^22^ Patients (*N* = 502) with severe AS scheduled to undergo TF‐TAVI with a balloon‐expandable transcatheter heart valve were enrolled from ten sites in Italy, the Netherlands, and the United Kingdom.^22^ FAST‐TAVI provides evidence that close monitoring, early mobilization and accelerated reconditioning, and discharge planning should be included in the TAVI program since these are important aspects of QoC.^19^ By adhering to the FAST‐TAVI discharge criteria, patients that were appropriately discharged early had a lower risk of TF‐TAVI‐related complications, such as all‐cause mortality, vascular complications (0.3% vs. 4.7%; *p* = .004), permanent pacemaker implantation (4.3% vs. 15.9%; *p* < .001), stroke (0.0% vs. 2.8%), and major bleeding at 30 days (0.3% vs. 6.5%; *p* < .001).^22^ The primary endpoint (a composite of all‐cause mortality, vascular access–related complications, permanent pacemaker implantation, stroke, cardiac rehospitalization, kidney failure, and major bleeding) was reached in 27% of patients (95% confidence interval [CI]: 23.3, 31.2) within 1‐year post‐procedure.^16^ Moreover, only 7.5% (95% CI: 5.5, 10.2) had in‐hospital complications before discharge and 19.6% (95% CI: 16.3, 23.4) within 1 year after discharge.^16^ This study highlights that adoption of simple, standardized TAVI‐specific QoC measures can help select patients for early discharge without impacting on clinical outcome.^21^, ^22^

The results of the European FAST‐TAVI project are supported by the outcomes of the French FAST‐TAVI 1 study,^13^, ^14^ which is a prospectively assess the feasibility and safety of within 72 hours discharge after transfemoral TAVI. Patients were prospectively assessed for early discharge home. Death or repeat hospitalization within 30 days occurred in 4 cases (5%) among patients discharged early. Factors associated with delayed discharge were blood transfusion (HR 13.85, 95% CI 1.61–119.40) and pacemaker implantation (HR 4.47, 95% CI 1.34–14.26). The authors confirmed the conclusion of the European FAST‐TAVI in that early discharge is safe and attainable in a large proportion of patients. The follow‐up study, FAST‐TAVI 2, is currently ongoing.

Data from the Vancouver 3M TAVR study also provides evidence that the BENCHMARK registry is feasible.^17^ Vancouver 3M is a prospective, multicenter study to document the efficacy, feasibility, and next‐day discharge of patients undergoing contemporary balloon‐expandable transfemoral TAVR using the minimalist Vancouver 3M Clinical Pathway approach. This clinical pathway was created to standardize TAVR care and reduce hospital stay length in a selected patient group; it includes measures such as risk‐stratified periprocedural practices, post‐procedure care, and a criteria‐driven discharge algorithm.^17^ Patients were screened (*N* = 1400) at 13 low‐, medium‐, and high‐volume North American centers between March 2015 and April 2017, of which 411 were enrolled with a median age of 84 years.^17^ The Vancouver 3M TAVR study results also demonstrated that a streamlined TAVI pathway allows for next‐day discharge home, with reproducible efficacy and safety outcomes.^17^ The composite primary endpoint of all‐cause mortality or stroke by 30 days occurred in 2.9% (95% CI: 1.7%, 5.1%) of patients.^17^ Notably, 80.1% of the elderly patients achieved next‐day discharge home, of which 89.5% were discharged home within 48 hours post‐procedure.^17^

Such is the importance of standardizing and streamlining care associated with the TAVI procedure that other countries and centers are reviewing possible initiatives, including the Canadian Cardiovascular Society Quality Initiative, the TAVI Care and Cure program developed in Rotterdam, and the European IMPULSE registry.^28^, ^29^, ^30^, ^31^ In addition, the guidelines from the European Society of Cardiology and the European Association for Cardio‐Thoracic Surgery support the QoC initiatives for patients undergoing TAVI, with the focus on patients being treated at heart valve centers/centers of excellence to deliver the best QoC for patients.^5^

The BENCHMARK registry will further support the findings from these studies, and determine potential cost savings and improvements in QoC that can be adopted as the new benchmark.

### 3.2 Potential limitations

The BENCHMARK registry will be conducted in multiple centers in seven European countries, which increases the applicability of findings but might limit the generalizability of the results across wider territories. Furthermore, not all patients having TAVI will be eligible for this registry. It is important to consider that inter‐ and intra‐country variation in healthcare systems and resources may have an unintended impact on the registry dataset. Due to the lack of central adjudication of safety events, the assessment of endpoints may be inconsistent between centers. This registry is not randomized and, as a result, there is the potential for confounding and bias in the analysis with limited ability for adjustment. In addition, we expect that length of stay will also decline in hospitals in general, but probably to a lesser extent. However, it is hoped that implementing standardized BENCHMARK QoC measures will minimize any bias owing to possible differences in healthcare systems.

## 4 CONCLUSIONS

Practice and patient outcomes in TAVI vary widely across TAVI centers in Europe and internationally, partly because QoC benchmarks for patient selection and discharge remains unclear. Several real‐world studies have already shown that streamlining care pathways, for example, appropriate early discharge home, can reduce resource utilization and improve patients' outcomes.^13^, ^14^, ^17^ The BENCHMARK registry will enable the next steps toward standardizing benchmark QoC measures in TAVI centers across Europe and worldwide.

## CONFLICT OF INTEREST

G. M. C., E. D., S. L., D. M., M. S., M. V. N., D. W., F. S. and D. F. received Honoria for consultancy from Edwards Lifesciences and are part of the BENCHMARK Steering Committee. C. M. L. and P. B. are representatives of the sponsor IPPMed who has received funding from Edwards Lifesciences. N. C. and J. K. are employees of Edwards Lifesciences.

## AUTHOR CONTRIBUTIONS

*Conception and design of the registry*: Gemma McCalmont, Eric Durand, Sandra Lauck, Douglas F. Muir, Mark S. Spence, Mariuca Vasa‐Nicotera, David Wood, Francesco Saia, Nicolas Chatel, Claudia M. Lüske, Jana Kurucova, Peter Bramlage, and Derk Frank. *Writing the first draft*: Gemma McCalmont, Claudia M. Lüske, Peter Bramlage. *Revising the article for important intellectual content*: Eric Durand, Sandra Lauck, Douglas F. Muir, Mark S. Spence, Mariuca Vasa‐Nicotera, David Wood, Francesco Saia, Nicolas Chatel, Claudia M. Lüske, Jana Kurucova, and Derk Frank. *Approving the article for submission*: Gemma McCalmont, Eric Durand, Sandra Lauck, Douglas F. Muir, Mark S. Spence, Mariuca Vasa‐Nicotera, David Wood, Francesco Saia, Nicolas Chatel, Claudia M. Lüske, Jana Kurucova, Peter Bramlage, and Derk Frank. *Taking over full responsibility*: Gemma McCalmont, Eric Durand, Sandra Lauck, Douglas F. Muir, Mark S. Spence, Mariuca Vasa‐Nicotera, David Wood, Francesco Saia, Nicolas Chatel, Claudia M. Lüske, Jana Kurucova, Peter Bramlage, and Derk Frank.

## Data Availability

This manuscript reports the rationale and design for the international BENCHMARK registry; no data are currently available.
